# Contribution of Oxidative Stress and Inflammation to the Neurogenic Hypertension Induced by Intermittent Hypoxia

**DOI:** 10.3389/fphys.2018.00893

**Published:** 2018-07-11

**Authors:** María P. Oyarce, Rodrigo Iturriaga

**Affiliations:** Laboratorio de Neurobiología, Facultad de Ciencias Biológicas, Pontificia Universidad Católica de Chile, Santiago, Chile

**Keywords:** carotid body, chronic intermittent hypoxia, hypertension, inflammation, nucleus of the solitary tract, pro-inflammatory cytokines

## Abstract

Chronic intermittent hypoxia (CIH), the hallmark of obstructive sleep apnea, is the main risk factor to develop systemic hypertension. Oxidative stress, inflammation, and sympathetic overflow have been proposed as possible mechanisms underlying the CIH-induced hypertension. CIH potentiates the carotid body (CB) chemosensory discharge leading to sympathetic overflow, autonomic dysfunction, and hypertension. Oxidative stress and pro-inflammatory molecules are involved in neurogenic models of hypertension, acting on brainstem and hypothalamic nuclei related to the cardiorespiratory control, such as the nucleus of the solitary tract, which is the primary site for the afferent inputs from the CB. Oxidative stress and pro-inflammatory molecules contribute to the activation of the CB chemoreflex pathway in CIH-induced hypertension. In this brief review, we will discuss new evidence for a critical role of oxidative stress and neuro-inflammation in development of the CIH-induced hypertension through activation of the CB chemoreflex pathway.

## Role of the Carotid Body in the CIH-Induced Hypertension

An abnormal heightened carotid body (CB) chemosensory discharge, which elicits sympathetic overflow, has been involved in the cardiovascular and autonomic alterations in preclinical models of human diseases such as obstructive sleep apnea (OSA), systolic heart failure, and neurogenic hypertension (Sun et al., 1999; Peng et al., 2003; Rey et al., 2004; Del Rio et al., 2010; Abdala et al., 2012; McBryde et al., 2013). The OSA syndrome characterized by repeated episodes of chronic intermittent hypoxia (CIH) is considered an independent risk factor for systemic hypertension, and is associated with atrial fibrillation, stroke, and heart failure (Somers et al., 2008; Dempsey et al., 2010). The cardiovascular consequences of OSA has been attributed to oxidative stress, inflammation, and sympathetic overflow induced by CIH, but other factors are influential, such as sleep fragmentation and co-morbid metabolic diseases (Gozal and Kheirandish-Gozal, 2008; Somers et al., 2008; Dempsey et al., 2010; Iturriaga et al., 2016; Iturriaga, 2017). OSA patients show enhanced sympathetic, vasopressor and ventilatory responses to hypoxia, attributed to a potentiated hypoxic peripheral chemoreflex (Somers et al., 2008; Dempsey et al., 2010). Similarly, rodents exposed to CIH show enhanced cardiorespiratory and sympathetic hypoxic responses, and develop hypertension (Fletcher et al., 1992; Peng et al., 2003; Iturriaga et al., 2009; Del Rio et al., 2010, 2014; Kumar and Prabhakar, 2012). Neural recordings of rat and cat CB chemosensory discharges have shown that CIH selectively increases the baseline discharge in normoxia and enhances the chemosensory responses to hypoxia (Peng et al., 2003; Rey et al., 2004; Del Rio et al., 2010). The enhanced CB chemosensory discharge induced by CIH has been linked with local oxidative stress and increased endothelin-1 (ET-1) levels in the CB (Peng et al., 2003; Rey et al., 2006; Del Rio et al., 2010). The enhanced CB chemosensory discharge plays a crucial role in the onset and progression of the hypertension induced by CIH. Indeed, Fletcher et al. (1992) found that CBs denervation prevents the hypertension in rats exposed to CIH. Furthermore, Del Rio et al. (2016) found that CBs ablation in hypertensive rats exposed to CIH for 21 days, restores the autonomic balance, the cardiac baroreflex sensitivity and reduces the elevated arterial pressure (BP), even when the CIH stimuli was maintained for 7 days and systemic oxidative stress persisted after the elimination of the CBs. Thus, the available evidence supports a crucial role of the CB in the onset and progression of the hypertension induced by CIH.

## CIH and CB Chemoreflex Potentiation

The CB chemoreceptor cells are innervated by sensory petrosal neurons that project to the nucleus of the tractus solitarius (NTS), which is the primary site of integration of gastrointestinal, respiratory and cardiovascular information in the brainstem (Berger, 1980; Finley and Katz, 1992; Grimes et al., 1995). The projections from the petrosal neurons that innervate the CB reach the caudal section of the NTS, specifically the dorsal, medial, and commissural sub-nuclei. In the NTS, second and third-order neurons project to the paraventricular nucleus (PVN) and the rostral ventrolateral medulla (RVLM), where are located the pre-sympathetic neurons (Guyenet, 2006). Hypoxia depolarizes chemoreceptor cells releasing excitatory transmitters, which in turn increases the frequency of discharge in the petrosal neurons eliciting reflex hyperventilatory, autonomic and vasopressor responses (Iturriaga and Alcayaga, 2004; Nurse and Piskuric, 2013). CIH enhanced the normoxic CB chemosensory discharge and the neural activity of the cardiorespiratory neurons in the brainstem and hypothalamus (Iturriaga et al., 2017). Indeed, CIH increases the electrical activity of glutamatergic neurons in the NTS (de Paula et al., 2007) and the number of c-fos or FosB positive neurons in the NTS, RVLM, PVN, in the subfornical organ (SFO) and median preoptic nucleus (Knight et al., 2011; Bathina et al., 2013; Sharpe et al., 2013). The activation of NTS and RLVM neurons induced by CIH is associated with local oxidative stress (Peng et al., 2014). Moreover, the increased Fos B in the RVLM induced by CIH (An and En-Shang, 2014) was attenuated by systemic pretreatment with a superoxide dismutase mimetic (Kuo et al., 2011). Thus, it is likely that the CIH-induced activation of the NTS and RVLM neurons is the result of oxidative stress (Daulatzai, 2012). Another plausible explanation is that the activation of the CB chemoreflex neural pathway triggered by the enhanced CB chemosensory discharge may elicit oxidative stress and neuroinflammation in the brainstem. This idea is strongly supported by the finding that CB neurotomy performed before the onset of CIH exposure prevents the oxidative stress in the NTS and RVLM, and the development of the hypertension in rats (Peng et al., 2014).

## Oxidative Stress and Inflammation in the CB and the Chemoreflex Neural Pathway Induced by Hypoxia

Reactive oxygen species (ROS) and reactive nitrogen species (RNS) contribute to enhance the CB chemosensory discharge and the progression of the hypertension in rats exposed to CIH (Prabhakar, 2000; Del Rio et al., 2010; Peng et al., 2014). Indeed, the treatment with antioxidants normalized the enhanced CB chemosensory discharge and prevents or reverses the elevated BP in CIH-treated rats (Peng et al., 2003, 2009; Del Rio et al., 2010; Moya et al., 2016).

In addition other molecules downstream the ROS signaling pathway may mediate the CIH-induced excitatory effects on CB chemoreception. Thus, we hypothesized whether pro-inflammatory molecules may contribute to enhance the CB chemosensory discharge (Iturriaga et al., 2009). Inflammation is part of the response of the immune system to tissue damage or pathogen invasion (Hänsel et al., 2010). The classical clinical signs of inflammation include increased blood flow, capillary permeability, release of inflammatory mediators and the migration of leukocytes (Hänsel et al., 2010). These processes are orchestrated by molecules activated by the nuclear transcription factor κB (NF-κB), which stimulates the release of pro-inflammatory cytokines such as tumor necrosis factor alpha (TNF-α), interleukin 1β (IL-1β) and interleukin 6 (IL-6), chemokines and adhesion molecules (Shih et al., 2015). The combination of cycles of hypoxia followed by re-oxygenation in OSA patients is associated with an increase of plasma levels of TNF-α, IL-6, and C-reactive protein (Meier-Ewert et al., 2004; Irwin et al., 2010). Most of the cellular responses and adaptations to hypoxia are mediated by the hypoxia-inducible factor-1α (HIF-1α) (Prabhakar and Semenza, 2012; Peng et al., 2014). NF-κB is a critical transcriptional activator of HIF-1α and it is necessary for the accumulation of HIF-1α during hypoxia (Hocker et al., 2017). On the other hand, hypoxia may directly activate the NF-κB factor, promoting the transcription of pro-inflammatory cytokines (Eltzschig and Carmeliet, 2011). Moreover, in response to oxidative stress, HIF-1α evokes the translocation of NF-κB to the nucleus increasing the expression of IL-1β, TNF-α, and ET-1 among other pro-inflammatory molecules (Chang et al., 2009). Zhang et al. (2015) studied the serum levels of inflammatory cytokines and the activation of NF-κB and HIF-1α in myocardial tissues in response to different frequencies of CIH (10–40 times/h for 6 weeks) and the actions of the antioxidant tempol. Intermittent hypoxia increased the serum levels of TNF-α, along with an increase on myocardial expression of NF-κB and HIF-1α in a frequency-dependent manner. Interesting, tempol treatment attenuated this effect (Zhang et al., 2015). Therefore, there is an interplay between oxidative stress, inflammation, and hypoxic induced factors under CIH conditions.

Chronic intermittent hypoxia increases the levels of pro-inflammatory molecules in the CB (Del Rio et al., 2011, 2012; Lam et al., 2012). Indeed, Rey et al. (2006) found that CIH increased ET-1 in the CB from cats exposed to CIH for 4 days, while bosentan reduced the CB chemosensory response to hypoxia *in vitro* in CIH-treated cats, but not in sham animals. Lam et al. (2012), reported that 7 days of exposure to CIH increased the mRNA levels of TNF-α, IL-6 and IL-1β, and their receptors in the rat CB. Moreover, Del Rio et al. (2012) found that exposure to CIH for 21 days produced a progressive increase of the immunoreactivity levels of TNF-α, IL-1β and iNOS in the rat CB, while ET-1 showed a transient increase during the first week of CIH. These results suggest that pro-inflammatory molecules may mediate the onset (ET-1) and the maintenance (pro-inflammatory cytokines) of the CB chemosensory potentiation. The treatment with ascorbic acid abolished the CIH-induced increases of TNF-α and IL-1β immunoreactivity levels in the CB, suggesting that inflammation depends on oxidative stress in the CB (Del Rio et al., 2012). The treatment with ibuprofen administered systemically during CIH did not reduce the enhanced CB chemosensory responses to hypoxia, although reduced the increased chemosensory baseline and the increased levels of pro-inflammatory cytokines in the CB (Del Rio et al., 2012). Nevertheless, the administration of ibuprofen prevents the hypertension induced by CIH exposure and the ventilatory acclimatization in rats, suggesting that ibuprofen may act in other sites of the chemoreflex pathway (Del Rio et al., 2012). Ibuprofen also prevents the increase in the number of *c-fos* positive neurons in the cNTS of rats subjected to CIH (Del Rio et al., 2012). Therefore, pro-inflammatory molecules may act on other neural structures of the CB chemoreflex pathway, such as the brainstem cardiorespiratory centers. Similarly, Popa et al. (2011), found in rats exposed to sustained hypoxia that the systemic administration of ibuprofen blocked the increase of IL-1β and IL-6 in the NTS and reduced the ventilatory response, indicating that these cytokines were crucial for the onset of the hyperventilatory response elicited by hypoxia (Popa et al., 2011). More recently, De La Zerda et al. (2017) tested the hypothesis that inflammatory signals are necessary to ventilatory acclimatization to sustained hypoxia applied for 11 days once it is established in rats. They found that hyperventilation was not affected by ibuprofen when was administered for the last 2 days of the hypoxic exposure (De La Zerda et al., 2017). In addition, they found that hypoxia (1 h) activated microglia in the NTS, effect that was abolished by ibuprofen administered from the beginning of hypoxic exposure (De La Zerda et al., 2017). The activation of the microglia induced by acute hypoxia lasted for 7 days, and was not altered by ibuprofen administered 2 days after the end of the hypoxia (De La Zerda et al., 2017). Thus, an early increase of pro-inflammatory molecules is required to produce hyperventilation following sustained hypoxia. Snyder et al. (2017) collected tissue punches from brain regions associated with different stages of neurodegenerative diseases in rats exposed CIH and measured oxidative stress and inflammatory markers. They found that CIH for 7 days produces oxidative stress and increases pro-inflammatory cytokines in brain areas associated to early stages of neurodegeneration (substantia nigra and entorhinal cortex) but not in the NTS and RVLM (Snyder et al., 2017). Our results agree with those observations. We found IL-1β, IL-6, and TNF-α mRNA levels were augmented in the NTS of hypertensive rats after 21 days of CIH (Oyarce and Iturriaga, 2018). These findings suggest that pro-inflammatory cytokines in the NTS may contribute to the maintenance of the hypertension, since CIH increases BP in 3–4 days in conscious rats, paralleling the time required to establish the enhanced CB chemosensory discharge (Del Rio et al., 2016).

## Neurogenic Hypertension and Inflammation

Neurogenic hypertension is associated with sympathetic overflow, increased plasma angiotensin II (Ang II) and C-reactive protein, TNF-α, IL-6, monocyte chemotactic protein 1, and adhesion molecules (Zubcevic et al., 2011), highlighting the importance of peripheral inflammation in hypertension. However, the role of central inflammation in neurogenic hypertension is gaining recognition. In the central nervous system, both circulating or released pro-inflammatory cytokines by astrocytes and microglia act on brainstem cardiovascular neurons (Shi et al., 2010). Waki et al. (2007) found that inducing inflammation in the NTS of normotensive rats by increasing the expression of the junctional adhesion molecule (JAM-1), triggers hypertension. They also compared the expression of JAM-1 in the NTS in young and adults spontaneously hypertensive rats (SHRs) and normotensive Wistar–Kyoto rats and found that JAM-1 mRNA was highly expressed in the NTS from SHR rats. Waki et al., 2010 using RT2 Profiler PCR arrays to detect changes on gene expression of cytokines and chemokines in the NTS from SHR, reported an abnormal expression of inflammatory mediators with relevant roles in the cardiovascular homeostasis, suggesting that cytokines may contribute to the hypertension by increasing the neuronal activity in the NTS (Waki et al., 2010). McBryde et al. (2013), reported that CB denervation reduces the number of CD3+ cells in the homogenate of the brainstem of SHR rats, suggesting that an enhanced CB chemosensory drive may induce the infiltration of T cells in brain tissues associated with the BP control (McBryde et al., 2013). The same group showed that systemic inflammation induced by LPS infusion activates the rat RVLM microglia, producing neuroinflammation and oxidative stress in rats, and neurogenic hypertension (Wu et al., 2012). In the PVN, the enhanced expression of pro-inflammatory cytokines elicits hypertension, while the blockade of TNF-α or NF-κB in the PVN attenuates the Ang-II-induced hypertension (Sriramula et al., 2013).

## Role of Microglia on Brain Inflammation and CIH

Activation of microglia, the brain resident macrophages, played a critical role in neuroplasticity and neuroinflammation (Shi et al., 2010; Bhalala et al., 2014; Kawabori and Yenari, 2015). Although ROS are essential for microglial inflammatory responses (Tschopp and Schroder, 2010), the specific involvement of microglia in CIH-induced neuroinflammation and hypertension is not completely known. Smith et al. (2013) found that 14 days of CIH increases the microglia mRNA expression of IL-1β, IL-6, COX-2 and the innate immune receptor TLR4 in the rat brainstem. Recently, Stokes et al. (2017), studied the role played by glial cells in the rat ventilatory acclimatization to sustained hypoxia. Using minocycline, an inhibitor of microglia activation with anti-inflammatory properties, they blocked both the microglial and astrocyte activation in the NTS and the ventilatory acclimatization of rats submitted to chronic hypoxia. One plausible mediator of the effects of CIH is Ang II, which induces microglial activation in the PVN and hypertension (Paton et al., 2008; Zhang et al., 2010). It is known that Ang II and pro-inflammatory cytokines molecules participates in the communication between neurons and glial cells (Kang et al., 2009).

## Role of the Circumventricular Organs in the Hypertension Induced by CIH

Circulating Ang II cannot effectively activate AT1 receptors (AT1R) in the NTS and RVLM of healthy subjects, because these receptors are protected by the brain barrier, but Ang II may access the brain through the circumventricular organs (CVOs), regions with weak brain barrier and a high density of AT1R (Banks and Erickson, 2010). Saxena et al. (2015) studied the contribution of Ang II on the sustained BP increase and FosB activation in the median preoptic and the PVN in rats with AT1R knockdown in the SFO. They found that CIH increased BP during the hypoxic exposure in both control and AT1R-knockdown rats. However, during the normoxic dark phase, only the controls showed a sustained BP elevation. AT1R-knockdown rats showed a decrease in the FosB mark in the median preoptic nucleus and the PVN. In addition, Kim et al. (2018) found that Ang II may act at the CB level. They found that the acute intermittent hypoxia-induced renal sympathetic overflow (RSO) was prevented by losartan. The CBs denervation and the pharmacological inhibition of the SFO produced a partial reduction of RSO, while combined CB denervation and SFO inhibition eliminated the increased sympathetic overactivity following intermittent hypoxia. Thus, the evidence suggests that SFO mediates the effects of elevated circulating levels of Ang II. Proinflammatory cytokines plays a key role in hypertension, but these molecules do not permeate the blood–brain barrier. Thus, it has been proposed that the CVOs mediate the hypertensive effects of circulating pro-inflammatory cytokines. Wei et al. (2013) found that the increased BP and RSA elicited by the intracarotid injection of TNF-α and IL-1β was attenuated in SFO-lesioned rats. They found that the increased BP and RSO induced by injections of TNF-α or IL-1β into the rat SFO were attenuated by microinjections of losartan and captopril in the SFO (Wei et al., 2015). More recently, Wei et al. (2018) found that the intravenous injection of IL-1β increased mRNA levels of the angiotensin-converting enzyme, AT1R, TNF-α, and IL-1β in the SFO and the PVN. Pretreatment with microinjections of losartan and captopril in the SFO attenuated the expression of these excitatory mediators in the SFO and in the PVN. These results show that pro-inflammatory cytokines increase renin–angiotensin activity and produce local inflammation in the SFO and PVN.

## Proposed Model for CIH Activation of the CB Chemoreflex Pathway

**FIGURE 1:**
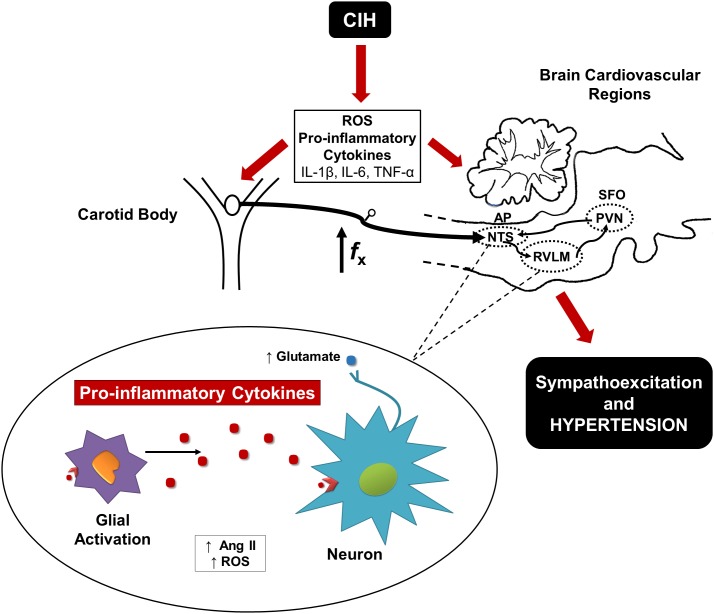
Proposed mechanism involved in CIH-induced hypertension. A diagram displays that the oxidative stress and pro-inflammatory molecules mediate the activation of the CB chemoreflex pathway leading to hypertension. In the NTS and RVLM, the hyperactivation of the neurons contributes to microglial activation increasing local levels of ROS, Ang II, and pro-inflammatory cytokines.

The available evidence indicates that the initial phase of the CIH-induced hypertension relays on the enhanced CB chemosensory drive, which triggers activation of neurons in the NTS and RVLM leading to hypertension (**Figure 1**). The CB is sensible to oxidative stress that contributes to potentiates the chemosensory discharge. As result of the activation of the neural pathway or a direct effect of CIH, the oxidative stress and pro-inflammatory molecules levels increase in the NTS and RVLM and contribute to the maintenance of hypertension. In addition, it is likely that circulating pro-inflammatory molecules and Ang II levels may enter the central nervous in the SFO and the AP (Simpson, 1981). The increased central activity may enhance the production of both ROS and pro-inflammatory cytokines in the NTS, which may induce microglial activation (Hirooka et al., 2010). At the same time, microglial activation may increase the neuronal expression of NF-ββ, which increase the production of pro-inflammatory cytokines (Shi et al., 2010). In late phases of CIH, the inflammatory state may contribute to increase the sympathetic activity leading to the production of more pro-inflammatory molecules (Fernandez et al., 2014). This positive feedback should result in a hyperactivation of RVLM and PVN neurons. In the NTS and RVLM, a positive feedback between ROS and Ang II may increase the activity of glutamatergic neurons that increase the excitatory sympathetic output to the kidneys, blood vessels, heart and adrenal gland, eliciting a sustained increase in the BP (Crowley, 2014).

## Author Contributions

MO and RI contributed equally to the manuscript and approved the final version.

## Conflict of Interest Statement

The authors declare that the research was conducted in the absence of any commercial or financial relationships that could be construed as a potential conflict of interest.
